# Lyme neuroborreliosis in Swedish children—PCR as a complementary diagnostic method for detection of *Borrelia burgdorferi* sensu lato in cerebrospinal fluid

**DOI:** 10.1007/s10096-020-04129-7

**Published:** 2021-01-02

**Authors:** Barbro H. Skogman, Peter Wilhelmsson, Stephanie Atallah, Ann-Cathrine Petersson, Katarina Ornstein, Per-Eric Lindgren

**Affiliations:** 1 Center for Clinical Research Dalarna – Uppsala University, Region Dalarna County, Falun, Sweden; 2 Faculty of Medicine and Health, Örebro University, Örebro, Sweden; 3 Division of Inflammation and Infection, Department of Biomedical and Clinical Sciences, Linköping University, Linköping, Sweden; 4 Department of Clinical Microbiology, Region Jönköping County, Jönköping, Sweden; 5 Department of infectious diseases, Sahlgrenska University Hospital, Gothenburg, Sweden; 6 Clinical Microbiology Laboratory, Laboratory medicine, Region Skåne, Lund, Sweden; 7 Ystad Hospital, Skåne University Health Care, Region Skåne, Ystad, Sweden; 8 Division of Clinical Microbiology, Laboratory Medicine, Ryhov County Hospital, Jönköping, Sweden

**Keywords:** Lyme neuroborreliosis, PCR, Children, Diagnosis, Diagnostic test

## Abstract

The aim of this study was to evaluate polymerase chain reaction (PCR) as a diagnostic method for the detection of *Borrelia burgdorferi* s.l. in CSF of Swedish children with LNB. This study was performed retrospectively on CSF and serum samples collected from children evaluated for LNB (*n* = 233) and controls with other specific neurological disorders (*n* = 59) in a Swedish Lyme endemic area. For anti-*Borrelia* antibody index, the IDEIA Lyme Neuroborreliosis kit (Oxoid) was used. Two in-house real-time PCR assays targeting the *16S* rRNA gene were evaluated (TaqMan® and LUX™). Among patients classified as LNB cases (*n* = 102), five children (5%) were *Borrelia* PCR-positive in CSF with the TaqMan® assay. In the Non-LNB group (*n* = 131), one patient was *Borrelia* PCR positive with the TaqMan® assay. Among controls (*n* = 59), all CSF samples were PCR negative. When amplifying and sequencing *ospA*, we found *B. garinii* (*n* = 2), *B. afzelii* (*n* = 2), *B. bavariensis* (*n* = 1), and one untypable (*n* = 1). With the LUX™ technology, all CSF samples were PCR negative. The TaqMan® assay could detect only few cases (*n* = 6) of *B. burgdorferi* s.l. in CSF among children with LNB and the sensitivity was very low (5%). However, using larger CSF volumes and centrifugation of samples, the PCR technique could still be useful as a complementary diagnostic method when evaluating LNB. Furthermore, detection of spirochete DNA in clinical matrices, including CSF, is the method of choice for studying epidemiological aspects of LNB, a tick-borne emerging disease.

## Introduction

Due to climate change and global warming, tick-borne diseases are spreading in the northern hemisphere [1–3]. Lyme borreliosis (LB) is the most common tick-borne infection reported in Northern America and Europe and it has been reported increasingly in the last 20 years [3, 4]. Because of this geographical spread of the infection and because of the national and regional awareness programs, LB is an increasing health issue, and the cause of many consultations at primary health centers, at pediatric and infectious diseases departments [5]. LB is caused by the spirochete *Borrelia* (*B*.) *burgdorferi* sensu lato (s.l.) [6]. In Europe, the species *B. afzelii*, *B. garinii*, *B. bavariensis*, and *B. burgdorferi* sensu stricto (s.s.) are all human pathogens, whereas in the USA, *B. burgdorferi* s.s. is the only species causing LB [7]. *Ixodes ricinus* is the predominant tick species in Sweden and the primary pathogen vector for both humans and animals [8, 9].

Lyme neuroborreliosis (LNB) in Europe is the second most frequent manifestation of LB after the skin manifestation erythema migrans (EM) [10, 11]. In children, LNB most commonly presents as a facial nerve palsy or a subacute meningitis [12–14], but cases with only non-specific symptoms (headache, fatigue) occur and may cause difficulties for the clinician [15, 16]. In some cases, LNB may be preceded by an EM in the head and neck region in the specific child [17]. Other major clinical manifestations of LB in children are lymphocytoma and Lyme arthritis [6].

There is no golden standard in the diagnostic tests of LNB, either in adults or in children.

The serological methods used in praxis for laboratory diagnostics in LNB are indirect methods [18–20]. They can demonstrate intrathecal production of anti-*Borrelia* antibodies, one important criterion for the LNB diagnosis [21], but they may also be negative early in LNB [22]. Other indirect diagnostic markers of LNB have also been studied, and the chemokine CXCL13 in CSF has shown promising results [23–27], also in pediatric LNB patients, where high sensitivity and specificity have been shown [23, 28]. Furthermore, an IL-10/CXCL1 ratio in CSF has been suggested to further improve LNB diagnostics in pediatric LNB patients [29]. As a direct detection method, culture of *Borrelia* spirochetes has been used, but the method has very low sensitivity and is technically difficult and tedious [30]. Detection of *Borrelia* spp. nucleic acids DNA in various clinical specimens, including the cerebrospinal fluid (CSF), has been tested in both adult and pediatric LNB patients, but sensitivity has been low [31–33]. Direct diagnostic methods based on polymerase chain reaction (PCR) are often “in-house” protocols and thus difficult to standardize [33]. A PCR assay targeting the *ospA* region and *rrf-rrl* region of the *Borrelia* genome detected LNB in adults with definite LNB and suspected LNB [31]. Furthermore, using PCR as a complementary diagnostic method for LNB patients with short duration of symptoms or LNB patients with negative *Borrelia* antibody index (AI) has been suggested [34, 35]. Additionally, identifying *Borrelia* spp. with PCR technology might be epidemiologically useful, since new causative agents for LNB in humans may emerge [2].

The aim of this study was to evaluate two different PCR technologies as diagnostic methods for direct detection of *B. burgdorferi* s.l. in CSF of well-characterized Swedish pediatric LNB patients.

## Material and methods

### Subjects

**Fig. 1:**
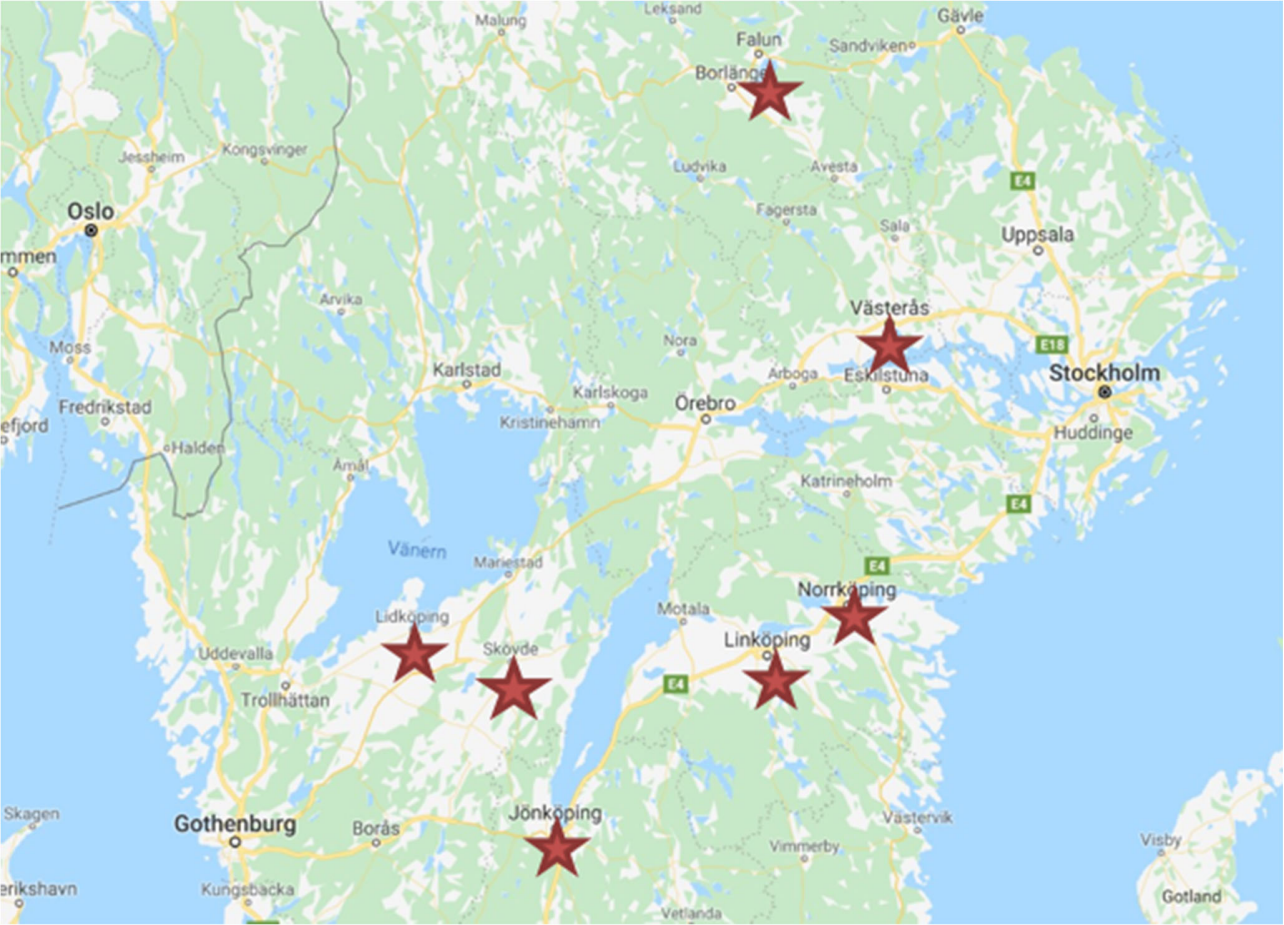
Map of the Lyme endemic region in Sweden where the study was conducted

This study was performed retrospectively on CSF and serum samples collected from children being evaluated for LNB (*n* = 233), and children with other specific neurological disorders (controls, *n* = 59) at seven pediatric departments in a Lyme endemic area in the central part of Sweden (Fig. 1), during the years 2011 to 2014.

All children were evaluated for LNB according to national guidelines, blood samples were drawn, and a diagnostic lumbar puncture was performed. CSF and serum samples were collected on admission, before the start of antibiotic treatment. Total cell counts in CSF were analyzed immediately at each local hospital laboratory, as part of the clinical routine for evaluation of pediatric LNB patients.

Non-centrifuged CSF samples (0.2–0.5 mL) were collected at the time of the diagnostic lumbar puncture and stored immediately at − 70 °C. Subsequent *Borrelia* spp. PCR analyses were performed at the Division of Inflammation and Infection, Linköping University, SE, and at the Clinical Microbiology Laboratory, Laboratory medicine, Region Skåne, SE.

Clinical and laboratory data were collected from standardized questionnaires and medical records. Questionnaires included clinical information about character and duration of symptoms, tick bites, and previous antibiotic treatment. A 2-month follow-up visit was carried out at each pediatric department to evaluate the clinical recovery.

### Classification of patients and controls

Children in the study were classified according to the European case-definitions for LNB (Table 1) [21]. All children in the Definite LNB (*n* = 68) and Possible LNB group (*n* = 34) received antibiotic treatment according to national guidelines (i.e., ceftriaxone i.v. 50–100 mg/kg once daily for 10–14 days for children < 8 years of age, and doxycycline p.o. 4 mg/kg once daily for 10–14 days for children ≥ 8 years of age). All patients in the Definite LNB and Possible LNB groups responded well to antibiotic treatment and are considered LNB patients. All LNB patients (*n* = 102) are used for calculation of sensitivity for the PCR assays.

**Table 1:** Classification of patients with Lyme neuroborreliosis (LNB) and controls

	Definite LNB (*n* = 68)	Possible LNB (*n* = 34)	Non-LNB (control group 1) (*n* = 131)	Other neurological disorders (control group 2) (*n* = 59)
Symptoms attributable to LNB	+	+	+	±
Pleocytosis in CSF	+	+	–	±
Anti-*Borrelia* AI	+	–	–	–

*LNB* Lyme neuroborreliosis, *CSF* cerebrospinal fluid, + yes, − no, *pleocytosis* total cell count > 5 × 10^6^/L in CSF, *AI* anti*-Borrelia* antibody index, positive if > 0.3, showing an intrathecal production of anti-*Borrelia* antibodies according to case-definition in European guidelines [21]

Children not meeting the criteria for Definite LNB or Possible LNB [21] (i.e., initially having symptoms suspected for LNB but with no pleocytosis in CSF and negative anti-*Borrelia* AI) were classified as Non-LNB (control group 1, *n* = 131). Additionally, another control group was included consisting of children with other specific neurological diagnoses (i.e., epilepsy, intracranial hypertension, metabolic diseases, viral meningitis, and encephalitis). This control group is referred to as Other neurological disorders (control group 2, *n* = 59).

All children in Non-LNB (control group 1, *n* = 131) and in Other neurological disorders (control group 2, *n* = 59) were negative for anti-*Borrelia* AI. Children in control group 2 (*n* = 59) are used for determination of specificity for the PCR assays.

### Laboratory methods

The serological method used for anti-*Borrelia* AI in this study, as part of clinical routine evaluation of the patient and for classification of patient groups, was the flagellin-based IDEIA Lyme neuroborreliosis kit (IgM and IgG) (Oxoid Limited, former DAKO, Hampshire, UK) [19]. Intrathecal production of anti-*Borrelia* antibodies (i.e., positive anti-*Borrelia* AI) was defined as AI > 0.3. Pleocytosis in CSF was defined as total white blood cell count > 5 × 10^6^/L in CSF [36]. In a few patients, CXCL13 data were available (recomBead CXCL13 assay, Mikrogen Diagnostik, Germany) [24].

Two different in-house real-time PCR assays, both targeting the *16S* rRNA gene of *Borrelia genus*, were evaluated for laboratory diagnosis of LNB in the present study. One PCR protocol included a real-time PCR assay (TaqMan® technology) targeting the *16S* rRNA region for *Borrelia* species and *B. burgdorferi* s.l., respectively, followed by a conventional nested PCR designed to amplify the *osp*A gene fragment, which would determine different *B. burgdorferi* s.l. species as described by Ornstein et al. [35, 37]. The other genus-specific real-time PCR assay was based on the Light Upon eXtension (LUX™) technology (Invitrogen Corporation, Paisley, United Kingdom) using 5 μL of template DNA, as previously described by Wilhelmsson et al. [9].

DNA was extracted from 200 μL CSF using Bio Robot EZ-1 with DNA Tissue Kit (Qiagen, Hilden, Germany) after treatment with Proteinase K at 56 °C for 30 min followed by heating at 95 °C for 15 min and addition of 0.01 μg calf thymus DNA (Sigma Aldrich). Elution volume was set to 50 μL. The amplification was carried out in a 25-μL reaction mix of SensiFAST Probe No-ROX Kit (Bioline, London, England) with primers and probes as described elsewhere, and 5-μL template [37–39].

In the TaqMan® real-time PCR protocol, *Borrelia* spp. were further determined by nucleotide sequencing the outer surface protein A gene (*osp*A) using a nested PCR approach as earlier described by Ornstein et al. [35]. PCR products were purified using a QIA quick PCR purification kit (Qiagen). Both strands of the approximately 200 base pair (bp) PCR product were sequenced using the BigDye Terminator Cycle Sequencing Kit (Applied Biosystems Inc., Foster City, CA, USA) and analyzed on an ABI PRISM 3100 Genetic Analyser (Applied Biosystems Inc.). A BLAST search was performed for all sequences [37].

All samples in the study (*n* = 292) were analyzed with the TaqMan® real-time PCR assay, but due to unforeseen technical issues, samples from only ninety-five patient samples (*n* = 95) were analyzed with the LUX™ real-time PCR assay.

## Results

### Clinical and laboratory characteristics

Out of 233 children being evaluated for LNB, 29% were classified as Definite LNB (*n* = 68), 15% as Possible LNB (*n* = 34), and 56% as Non-LNB (control group 1, *n* = 131). Clinical signs and symptoms and laboratory data in different groups are shown in Table 2. The group of Other neurological disorders (control group 2, *n* = 59) consisted of children with epilepsy, infantile spasm, high intracranial pressure, head trauma, papilledema, viral encephalitis, aseptic meningitis, varicella infection, myasthenia gravis, and the Guillain-Barre Syndrome. Clinical characteristics in all patient groups are shown in Table 2.

**Table 2:** Clinical and laboratory characteristics of the children of the different groups

	Definite LNB *n* = 68		Possible LNB *n* = 34		Non-LNB *n* = 131		Other neurological disorders *n* = 59	
Gender								
Female/male, *n* (%)	30/38	(44/56)	15/19	(44/56)	82/49	(63/37)	29/30	(49/51)
Age, median (range, years)	8	(2–15)	8	(4–14)	10	(1–18)	10	(0–17)
Observed tick bites, *n* (%)	41	(60)	18	(53)	58	(44)	15	(25)
Clinical characteristics								
Erythema migrans, *n* (%)	28	(41)	9	(26)	18	(14)	2	(3)
Lymphocytoma, *n* (%)	5	(7)	3	(9)	7	(5)	0	(0)
Facial nerve palsy, *n* (%)	46	(68)	26	(76)	51	(39)	4	(7)
Headache, *n* (%)	49	(72)	23	(68)	90	(69)	40	(68)
Fatigue, *n* (%)	62	(91)	22	(65)	87	(66)	36	(61)
Fever, *n* (%)	37	(54)	11	(32)	23	(18)	20	(34)
Neck pain, *n* (%)	36	(53)	17	(50)	34	(26)	19	(32)
Neck stiffness, *n* (%)	23	(34)	11	(32)	18	(14)	12	(20)
Loss of appetite, *n* (%)	43	(63)	18	(53)	45	(34)	19	(32)
Nausea, *n* (%)	24	(35)	11	(32)	45	(34)	24	(41)
Vertigo, *n* (%)	10	(15)	6	(18)	56	(43)	25	(42)
Other symptoms, *n* (%)	48	(71)	17	(50)	50	(38)	39	(66)
Duration of neurological symptoms								
1–2 days, *n* (%)	5	(7)	6	(18)	16	(12)	5	(8)
3–6 days, *n* (%)	26	(38)	15	(44)	22	(17)	10	(17)
1–2 weeks, *n* (%)	21	(31)	5	(15)	12	(9)	3	(5)
3–4 weeks, *n* (%)	11	(16)	4	(12)	12	(9)	5	(8)
1–2 months, *n* (%)	1	(1)	0	(0)	7	(5)	3	(5)
> 2 months, *n* (%)	1	(1)	2	(6)	30	(23)	9	(15)
Unknown, *n* (%)	3	(4)	2	(6)	32	(24)	24	(41)
Clinical recovery within 2 months, *n* (%)	58	(85)	28	(82)	99	(76)	36	(61)
Laboratory findings								
Total number of cells × 10^6^/L in CSF, median (range)	147	(20–890)	141	(8–486)	0	(0)	138	(6–1125)
Mononuclear cells × 10^6^/L in CSF, median (range)	140	(12–885)	134	(0–484)	0	(0)	134	(6–1125)
Anti-Borrelia IgM antibodies in CSF, *n* (%)	50	(74)	0	(0)	0	(0)	0	(0)
Anti-Borrelia IgG antibodies in CSF, *n* (%)	50	(74)	1	(3)	0	(0)	0	(0)
Anti-Borrelia IgM antibodies in serum	32	(47)	19	(56)	15	(11)	1	(2)
Anti-Borrelia Ig G antibodies in serum, *n* (%)	42	(62)	13	(38)	18	(14)	5	(8)
Anti-Borrelia AI, *n* (%)	68	(100)	1	(3)	0	(0)	0	(0)
Borrelia PCR (TaqMan® technology), *n* (%)	4	(6)	1	(3)	1	(1)	0	(0)
Borrelia PCR (LUX™ technology), *n* (%)	0	(0)	0	(0)	0	(0)	0	(0)

*LNB* Lyme neuroborreliosis, *CSF* cerebrospinal fluid, *PCR* polymerase chain reaction, *AI* anti*-Borrelia* antibody index is positive if > 0.3, showing an intrathecal production of anti-*Borrelia* antibodies according to case-definition in European guidelines [21]

### Detection of *Borrelia* species by two different PCR assays

Among patients classified as Definite LNB (*n* = 68) and Possible LNB (*n* = 34), only five children (5%) were *Borrelia* spp. PCR positive in CSF with the TaqMan® assay (Table 2). Additionally, in the Non-LNB group (*n* = 131), one patient was *Borrelia* PCR positive in CSF with the TaqMan® assay (Table 2). When amplifying and sequencing the OspA gene, the species found were *B. garinii* (*n* = 2), *B. afzelii* (*n* = 2), and *B. bavariensis* (*n* = 1), and in one sample, the *Borrelia* species was untypable (*n* = 1) (Table 3).

**Table 3:** Overview over *Borrelia* PCR-positive patients

	Case 1	Case 2	Case 3	Case 4	Case 5	Case 6
*Borrelia burgdorferi* species	*Borrelia garinii*	*Borrelia garinii*	*Borrelia afzelii*	*Borrelia afzelii*	*Borrelia bavariensis*	*Untypable*
Gender	Male	Female	Male	Female	Female	Female
Age (years)	11	5	4	12	6	6
Observed tick bite	+	–	+	–	–	–
Month of lumbar puncture	June	October	December	March	July	October
Clinical characteristics						
Erythema migrans/lymphocytoma	*–*	*+*	*–*	*–*	*–*	*–*
Facial nerve palsy	*–*	*+*	*+*	*+*	*+*	*+*
Headache	*+*	*+*	*+*	*+*	*+*	*+*
Fatigue	*+*	*+*	*+*	*+*	*+*	*+*
Other symptoms	*+*	*+*	*–*	*–*	*+*	*+*
Duration of neurological symptoms	7–14 days	3–6 days	3–6 days	3–6 days	1–2 days	3–6 days
Clinical recovery within 2 months	+	+	+	+	+	+
Laboratory findings						
Pleocytosis in CSF	+	+	+	–	+	+
Total cell count in CSF, *n*	374	220	28	0	164	278
Mononuclear cells in CSF, *n*	366	210	27	0	158	262
Anti-Borrelia IgM in CSF	*+*	*+*	*–*	*–*	*+*	*–*
Anti-Borrelia IgG in CSF	*–*	*–*	*+*	*–*	*–*	*–*
Anti-Borrelia IgM in serum	*–*	*–*	*–*	*+*	*+*	*+*
Anti-Borrelia IgG in serum	*–*	*+*	*–*	*–*	*–*	*+*
Anti-Borrelia AI	*–*	*+*	*+*	*–*	*+*	*–*
CXCL 13 in CSF (pg/mL)	*NA*	*NA*	710	9	46,600	6060
Borrelia PCR (TaqMan®)	*+*	*+*	+	+	+	+
Initial diagnosis	Possible LNB	Definite LNB	Definite LNB	Non-LNB	Definite LNB	Possible LNB
Re-defined diagnosis	Definite LNB	Definite LNB	Definite LNB	Definite LNB	Definite LNB	Definite LNB

*LNB* Lyme neuroborreliosis, *CSF* cerebrospinal fluid, *PCR* polymerase chain reaction, + yes, − no, *NA* not available, *pleocytosis in CSF* total cell count > 5 × 10^6^/L in CSF. *CXCL 13 in CSF* with recomBead assay with cut-off 160 pg/mL), *AI anti-Borrelia antibody index* is positive if > 0.3, showing an intrathecal production of anti-*Borrelia* antibodies according to case-definition in European guidelines [21]

Out of the six *Borrelia* PCR-positive patients, four patients had initially been classified as Definite LNB, one patient as Possible LNB, and one patient as Non-LNB (Table 3), according to the European guidelines [21]. The PCR-positive patients had clinical symptoms attributable to LNB such as fever, headache, fatigue, and/or facial nerve palsy. One of them, diagnosed as Definite LNB, also had a skin manifestation with a probable lymphocytoma (*B. garinii* PCR positive) (see description of cases below). At the 2-month follow-up visit, all six *Borrelia* PCR-positive patients reported full clinical recovery, even the one patient classified as Non-LNB, who did not receive antibiotic treatment. Thus, the diagnostic performance for the TaqMan® protocol showed a clinical sensitivity of 6% in the Definite LNB group (i.e., four positive PCR out of 68 patients) and 3% in the Possible LNB group (i.e., one positive PCR out of 34 patients). The overall clinical sensitivity was 5% (i.e., five positive PCR out of 102 patients in the Definite LNB and Possible LNB groups together). The clinical specificity was 100% (i.e., no positive PCR with the TaqMan® protocol among 59 patients in the group of Other neurological disorders). The one patient with positive PCR in Non-LNB was a misdiagnosed LNB patient at the time of routine laboratory evaluation and therefore received no antibiotic treatment.

Among 95 patients analyzed with the LUX™ real-time *Borrelia* PCR protocol, all of them were negative with the LUX™ *Borrelia* PCR assay (Table 2). They were classified as Definite LNB (*n* = 24), Possible LNB (*n* = 18), Non-LNB (*n* = 41), and Other neurological disorders (*n* = 12).

### Description of cases

The six *Borrelia* PCR*-*positive children are shown in Table 3 and described in more detail below.

#### Case 1

The first case is an 11-year-old boy with headache, fatigue, neck pain, neck stiffness, and vertigo for 1–2 weeks, June 2011. He had observed a tick bite 2–4 weeks before, but no EM. He was living in Jönköping but had been in the County of Östergötland and on the island of Öland during the summer. He had pleocytosis in CSF (total number of cells 374 × 10^6^/L, out of which 366 were mononuclear cells), positive anti-*Borrelia* IgM antibodies in serum but negative anti-*Borrelia* AI. Data on CXCL13 in CSF was not available. He was diagnosed as Possible LNB and was treated with doxycycline perorally for 14 days. He reported as full recovered at the clinical follow-up. He was PCR positive for *B. garinii* in CSF so his diagnosis was re-defined as Definite LNB.

#### Case 2

The second case is a 5-year-old girl with facial nerve palsy, fatigue, headache, fever, neck pain, and loss of appetite for 3–6 days in October 2011. She had not noticed any tick bites, but probably a redness of the earlobe (lymfocytoma) and also in the face (EM). She was living in the County of Östergötland (Linköping) but during summer had visited Stockholm (east cost of Sweden) as well as Gothenburg (west coast of Sweden). She had pleocytosis in CSF (total number of cells 220 × 10^6^/L, out of which 210 were mononuclear cells) and positive anti-*Borrelia* AI. Data on CXCL13 in CSF was not available. She was treated with ceftriaxone intravenously for 10 days. She had not reported persisting problems, but did not come for the final follow-up. She was PCR positive for *B. garinii* in CSF. She was initially classified as Definite LNB and remained so.

#### Case 3

The third case was a 4-year-old boy with facial nerve palsy, headache, and fatigue for 3–6 days. He was admitted in December 2012. He had noticed a tick bite 3–5 months before, but no EM. He was living in Norrköping in the County of Östergötland and had visited the island of Öland (east coast of Sweden) during summer. He had pleocytosis in CSF (total number of cells 28 × 10^6^/L, out of which 27 were mononuclear cells), a positive anti-*Borrelia* AI, and positive CXCL13 in CSF (710 pg/mL). He was treated with ceftriaxone intravenously for 10 days and was fully recovered at follow-up. He was PCR positive for *B. afzelii* in CSF. His diagnosis was initially and remained Definite LNB**.**

#### Case 4

The forth case is a 12-year-old girl with facial nerve palsy, headache, and fatigue for 3–6 days. She was admitted in March 2012. She had noticed neither tick bite nor EM. She was living in Skövde in Skaraborg area (central southern Sweden) but had visited the west coast of Sweden during summer. She had no pleocytosis in CSF, negative anti-*Borrelia* AI, and negative CXCL13 in CSF (9 pg/mL). She was initially treated with cortisone as in idiopathic facial nerve palsy, but due to positive anti-*Borrelia* IgM antibodies in serum, she was additionally treated with doxycycline perorally for 10 days. She was completely recovered at the clinical follow-up already 2 weeks later. Her diagnosis was initially idiopathic facial nerve palsy as part of the group Non-LNB. However, since she was PCR positive for *B. afzelii* in CSF; she was re-defined as Definite LNB, having a very early LNB with cranial nerve affection, but not yet pleocytosis in CSF.

#### Case 5

This was a 6-year-old girl with facial nerve palsy for 1–2 days, headache, and fatigue since 1–2 weeks and who was admitted in July 2014. She had also pain in the jaws, neck, and scalp. Tick bites or an EM had not been observed. This girl was living in Falun in the County of Dalecarlia and had been visiting Stockholm and Uppsala (east part of Sweden). She had pleocytosis in CSF (total number of cells 164 × 10^6^/L, out of which 158 were mononuclear cells), positive anti-*Borrelia* AI, and highly positive CXCL13 in CSF (46,600 pg/mL). She was treated with ceftriaxone intravenously for 14 days. She recovered without any sequelae. She was PCR positive for *B. bavariensis* in CSF. Her diagnosis was initially and remained Definite LNB.

#### Case 6

The last case was a 6-year-old girl admitted to the outpatient pediatric department in Linköping in October 2011 because of facial nerve palsy for 1–2 days, headache, and fatigue for 3–6 days. No tick bite had been noticed and she had not developed any EM. She was living in the County of Östergötland and had not been visiting other parts of Sweden. She had pleocytosis in CSF (total number of cells 278 × 10^6^/L, of which 262 were mononuclear cells), negative anti-*Borrelia* AI, and positive CXCL13 in CSF (6060 pg/mL). In serum, she had elevated titers of anti-*Borrelia* IgM and IgG antibodies. She was diagnosed as Possible LNB and received ceftriaxone intravenously for 10 days with good clinical effect. Her symptoms had resolved already at the 3-week follow-up. She was PCR positive for *Borrelia*, but the species in this last case was untypable. Her diagnosis was re-defined as Definite LNB.

## Discussion

In this study we have evaluated two in-house real-time PCR protocols, both based on amplification of the *16S* rRNA gene fragment, in CSF of pediatric LNB patients and controls. The first protocol was a real-time PCR assay for the detection of the *16S* rRNA of *Borrelia* spp. (TaqMan®) followed by a nested *osp*A PCR. The second *16S* r RNA PCR assay was based on LUX technology. We had expected an acceptable sensitivity in children with short duration of symptoms (i.e., early LNB cases), especially in children with a high clinical suspicion of LNB, pleocytosis in CSF but negative anti-*Borrelia* AI (Possible LNB). However, with the TaqMan® protocol, *B. burgdorferi* s.l. DNA could be detected only in six out of 233 samples, whereas with the LUX™ protocol, all 95 tested samples were negative. Thus, the clinical sensitivity for PCR in children with LNB remains low with an overall clinical sensitivity of 5% (in the Definite LNB and Possible LNB groups taken together). Our PCR results indicate that one patient in the Possible LNB group should be re-defined as Definite LNB and one patient in the Non-LNB should be re-defined as Definite LNB (having a very early LNB with cranial nerve affection, but not yet pleocytosis in CSF). This supports the usefulness of a PCR protocol (TaqMan® assay) as a complementary diagnostic method for detection of *B. burgdorferi* s.l. in CSF in pediatric LNB patients with short duration of symptoms. The specificity of the TaqMan® protocol was as high as expected (100%).

Our findings of low sensitivity in CSF of patients with LNB are in concordance with previous studies [39, 40]. However, in a recent study from Norway, a much higher sensitivity in pediatric LNB patients is reported with a *Borrelia* PCR positivity of 46% in CSF of patients defined as Definite LNB and 50% in CSF of patients defined as Probable LNB (i.e., symptoms attributable to LNB, pleocytosis in CSF, negative AI but presence of anti-*Borrelia* antibodies in either CSF or serum) [34]. However, it is interesting to note that none of the patients in the Possible LNB group in the Norwegian study was PCR positive (i.e., patients with clinical symptoms attributable to LNB, lymphocytic pleocytosis in CSF, negative anti-*Borrelia* AI, and negative anti-*Borrelia* antibodies in serum) [34]. Duration of neurological symptoms was relatively short and comparable to symptom duration in LNB patients in our present study.

The reason for discordance in sensitivity between different studies might reasonably be found in the primer/probe design of the different PCR assays. However, the two PCR protocols used in the present study (the TaqMan® and the LUX™, respectively) have previously been compared in another study and they have shown very similar results [39].

It has been suggested that the volume and procedure of collected CSF could play a role in the probability of detecting the *B. burgdorferi* s.l DNA since the number of *Borrelia* spirochetes in CSF is suspectedly very low [41]. In our present study, a CSF volume of 0.2–0.5 mL (not centrifuged) was used for *Borrelia* PCR detection, whereas in the Norwegian study, CSF volumes between 0.5–1 mL were centrifuged into pellets and resuspended in 0.2 mL of CSF. Thus, this difference in CSF volume and procedure may be the major explanation for the discrepancy between PCR results. Consequently, larger volumes of CSF samples, improved procedure of centrifuging, lower elution volume (< 30 μL) or larger volumes of DNA templates (> 5 μL) should, if possible, be used when evaluating pediatric LNB patients, in order to obtain a higher proportion of *Borrelia* PCR-positive LNB patients.

Three major *B. burgdorferi* species have been detected in CSF of Swedish LNB patients: *B. burgdorferi* s.s., *B. afzelii*, and *B. garinii* [35]. In ticks, the incidence of *B. afzelii* predominates, followed by *B. garinii* and *B. burgdorferi* s.s. [9]. In the study from Norway, *B. garinii* was detected in CSF from 16 out of 35 LNB cases, whereas the rest could not be completely identified [34]. *Borrelia bavariensis* has, to our knowledge, not previously been found in CSF in pediatric LNB patients. The clinical picture of our LNB patient with positive PCR for *B. bavariensis* is very much representative of a typical pediatric LNB case (Table 3). Since *B. bavariensis* and *B. garinii* are phylogenetically very similar, they may have not been distinguished from each other in previous studies. Our result provides therefore a new interesting epidemiological aspect of pediatric LNB infection [34, 42].

Furthermore, in the present study in which *B. burgdorferi* s.l. species were identified by amplifying and sequencing the *osp*A gene, one *Borrelia*-positive sample could not be identified to species. One reason for this could be a low amount of *Borrelia* spirochetes in the initial CSF sample taken from the patient. Another reason could be that the detected *Borrelia* spirochete lacks the plasmid containing ospA. Previous studies have shown that PCR-methods for detecting *osp*A are unable to detect the species *B. spielmanii* and *B. miyamotoi*, both of which are known to be pathogenic to humans [39].

Facial nerve palsy was the main specific neurological manifestation in five out of six *Borrelia* PCR-positive LNB cases, where as in one child (Case 1), symptoms were less specific with headache, fatigue, neck pain/stiffness, and vertigo. This finding suggests that detectable levels of *Borrelia* DNA in the CSF are associated with both specific and non-specific neurological symptoms in LNB, which may be relevant but challenging knowledge for the clinician. Furthermore, high levels of pleocytosis in CSF were found in the two *B. garinii*-infected LNB patients and in the one LNB patient with *B. bavariensis.* These two species are known to induce a prominent inflammatory response, whereas *B. afzelii* infections are associated with a lower level of inflammation in the CSF [35, 40].

One patient with *B. afzelii* (Case 4), who was primarily classified as Non-LNB (due to absence of pleocytosis and negative anti-*Borrelia* AI), was, in our present study, re-defined as Definite LNB due to positive *Borrelia* PCR in CSF. This case was a very early case of LNB with neither pleocytosis nor intrathecal production of anti-*Borrelia* antibodies present, but with a positive serum IgM. This case shows us that in very early LNB cases, children might start producing *Borrelia* antibodies in serum before showing any sign of inflammation in CSF. Detection of *Borrelia* infection in the early phase by positive *Borrelia* PCR has been previously described [34, 41].

## Conclusions

The TaqMan® protocol could detect a few cases (*n* = 6) of *B. burgdorferi* s.l. in CSF among children with LNB, but the overall sensitivity was very low (5%). As methodological aspects could be improved, using larger CSF volumes and to centrifuge all CSF samples, the PCR technique could still be useful as a complementary diagnostic method, when evaluating pediatric LNB cases with short duration of neurological symptoms. Furthermore, detection of spirochete DNA in various clinical matrices, including CSF, is still the method of choice for studying epidemiological aspects of LNB, a tick-borne emerging disease.

## Data Availability

Data will be made available, if appropriate, after contact with first author.
